# CT and MRI imaging features of atypical spindle cell lipomatous tumor in the retroperitoneum: a case report

**DOI:** 10.3389/fonc.2026.1796519

**Published:** 2026-09-29

**Authors:** XueRong Li, Gang Li

**Affiliations:** Department of Radiology, The First Affiliated Hospital of Shandong First Medical University & Shandong Provincial Qianfoshan Hospital, Jinan, Shandong, China

**Keywords:** atypical spindle cell lipomatous tumor, computed tomography, differential diagnosis, magnetic resonance imaging, retroperitoneum

## Abstract

Atypical spindle cell lipomatous tumor (ASCLT) is a rare benign adipocytic neoplasm, and primary involvement of the retroperitoneum is exceedingly uncommon. Because its imaging features overlap with those of other fat-containing tumors, particularly atypical lipomatous tumor/well-differentiated liposarcoma (ALT/WDL), preoperative diagnosis remains challenging. We report a case of primary retroperitoneal ASCLT in a 58-year-old woman whose lesion was incidentally detected during a routine physical examination. Computed tomography (CT) and magnetic resonance imaging (MRI) revealed a small, well-defined fat-containing mass located between the celiac trunk and the superior mesenteric artery, with fat-related signal attenuation on fat-suppressed sequences, no obvious diffusion restriction, and mild reticular enhancement after contrast administration. Complete surgical excision was performed, and postoperative histopathological, immunohistochemical, and molecular findings supported the diagnosis of ASCLT, with no MDM2 amplification. Compared with previously reported retroperitoneal ASCLT cases, the present case provides detailed CT and multiparametric MRI findings for a small, incidentally detected lesion adjacent to major abdominal vessels. Recognition of this imaging pattern may help include ASCLT in the preoperative differential diagnosis and support appropriate surgical planning, while definitive diagnosis still requires pathological and molecular confirmation.

## 1 Introduction

Atypical spindle cell lipomatous tumor (ASCLT) is a rare benign adipocytic neoplasm with locally recurrent potential but no metastatic potential. In the 2020 World Health Organization (WHO) Classification of Tumors of Soft Tissue and Bone, atypical spindle cell/pleomorphic lipomatous tumor (ASCPLT) is recognized as a distinct benign adipocytic tumor rather than as part of the atypical lipomatous tumor/well-differentiated liposarcoma (ALT/WDL) spectrum (1). ASCLT most commonly arises in the superficial soft tissues of the extremities and trunk, whereas primary involvement of the retroperitoneum is exceedingly rare (2, 3). Because its imaging features can overlap with those of ALT/WDL and other lipogenic tumors, accurate preoperative diagnosis remains challenging. Previously published retroperitoneal cases have usually presented as enlarging or very large masses suspected preoperatively to be liposarcoma (3–5). We present a smaller, incidentally detected primary retroperitoneal ASCLT treated surgically at The First Affiliated Hospital of Shandong First Medical University, with emphasis on CT and multiparametric MRI findings, imaging-pathologic correlation, and the potential influence of preoperative recognition on surgical planning. The diagnostic and treatment timeline is shown in **Figure 1**.

**Figure 1:**
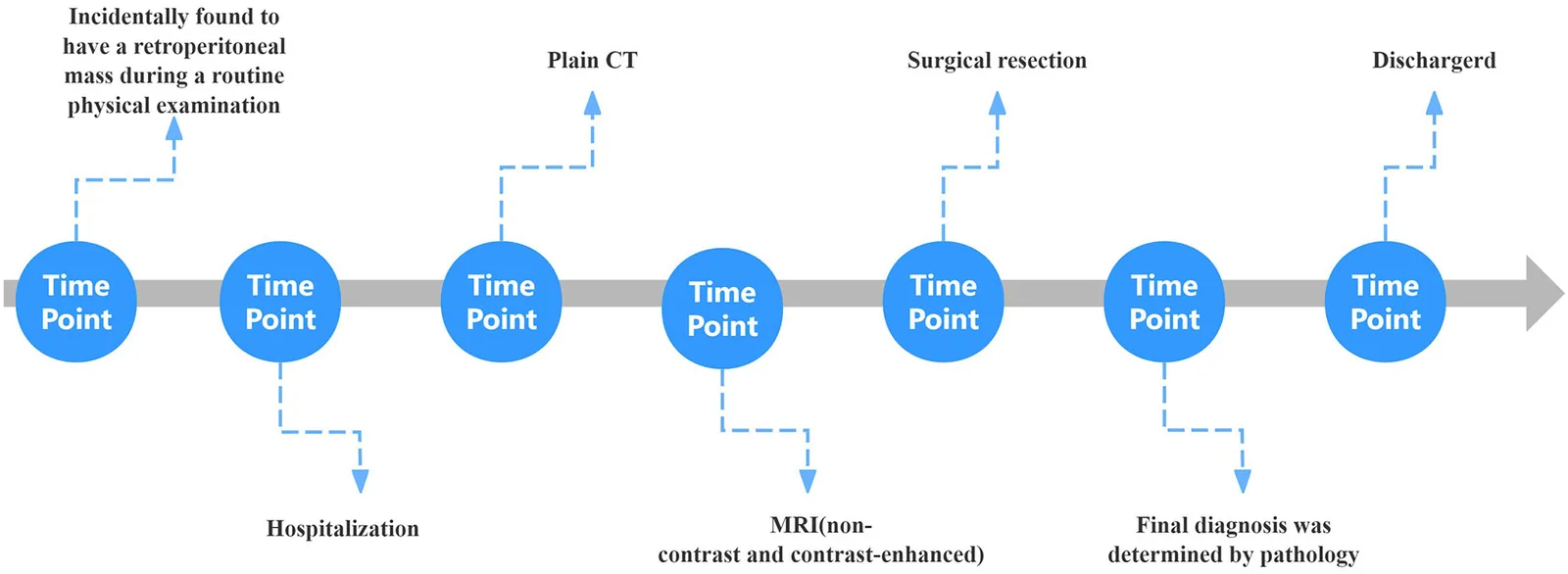
Diagnostic and treatment timeline of the patient.

## 2 Case description

A 58-year-old woman was admitted to our hospital after a retroperitoneal mass had been incidentally detected during a physical examination at an outside institution one month earlier. The patient was asymptomatic, and her medical history was unremarkable. Laboratory examinations, including liver and renal function tests and complete blood count, revealed no abnormalities. To further characterize the lesion, unenhanced abdominal computed tomography (CT) and magnetic resonance imaging (MRI), including contrast-enhanced sequences, were performed.

CT findings: Unenhanced CT showed a patchy, slightly hypoattenuating lesion anterior to the abdominal aorta, measuring approximately 2.8 cm x 2.0 cm. Focal fat attenuation was visible within the lesion (**Figures 2A, B**).

**Figure 2:**
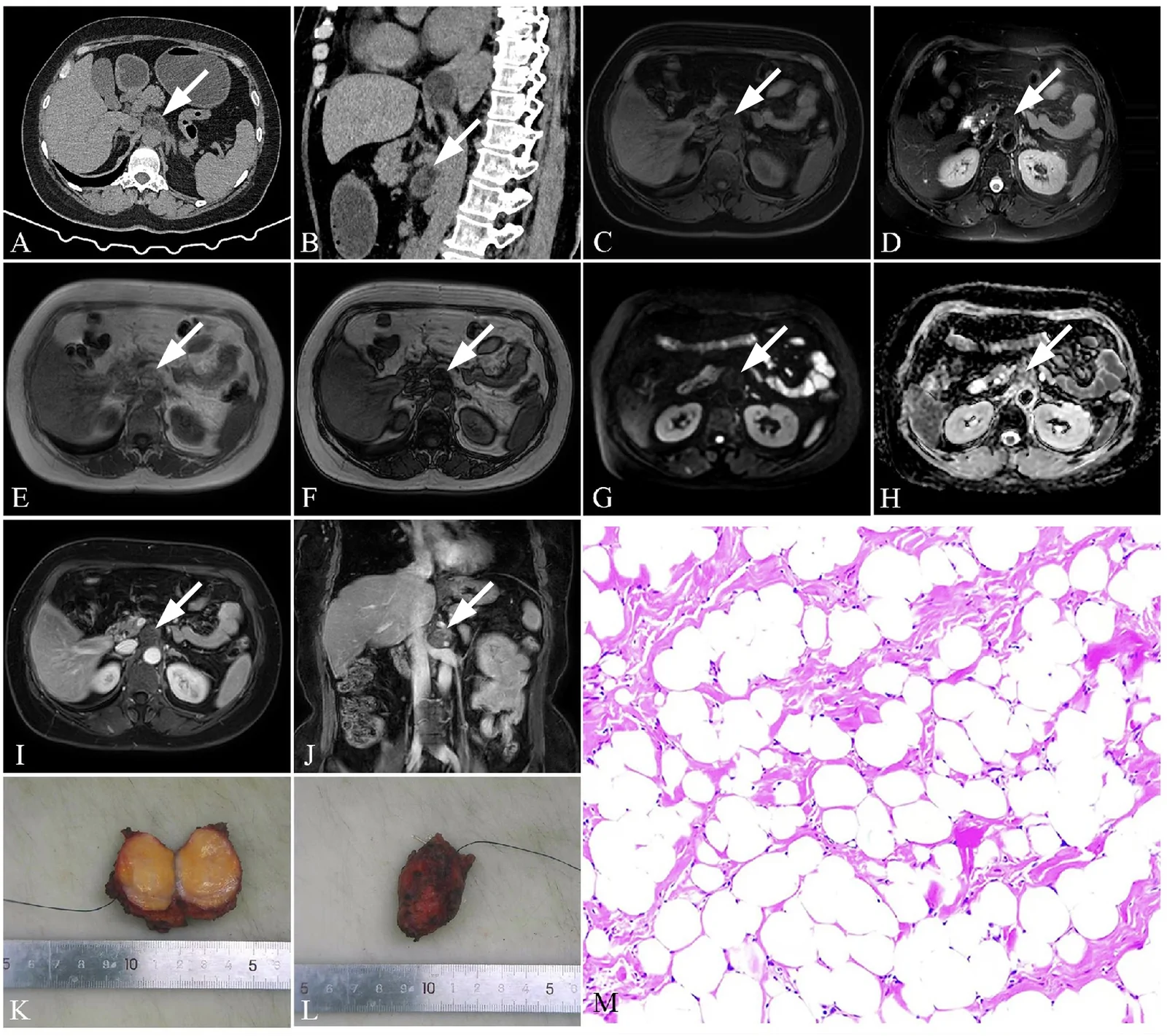
**(A, B)** Unenhanced computed tomography images show a small, slightly hypoattenuating retroperitoneal lesion anterior to the abdominal aorta, with focal fat attenuation. **(C, D)** Magnetic resonance imaging shows a well-defined retroperitoneal mass between the celiac trunk and the superior mesenteric artery, with iso- to slightly hypointense signal intensity on T1-weighted imaging and iso- to slightly hyperintense signal intensity on T2-weighted fat-suppressed imaging. **(E, F)** In-phase and opposed-phase images further suggest the presence of intralesional lipid components. **(G, H)** Diffusion-weighted imaging and the corresponding apparent diffusion coefficient map show no obvious diffusion restriction. **(I, J)** Contrast-enhanced axial and coronal magnetic resonance images demonstrate mild reticular enhancement of the lesion and its close relationship with adjacent major vessels. **(K, L)** Gross pathological images show a relatively well-circumscribed, yellowish tumor. **(M)** Hematoxylin and eosin staining shows mature adipose tissue admixed with spindle cells and collagenous/fibromyxoid stroma, supporting the diagnosis of atypical spindle cell lipomatous tumor.

MRI findings: MRI demonstrated a well-defined, round retroperitoneal mass between the celiac trunk and the superior mesenteric artery, measuring approximately 2.2 cm x 2.7 cm on axial images. The lesion showed iso- to slightly hypointense signal intensity on T1-weighted imaging (T1WI) and iso- to slightly hyperintense signal intensity on T2-weighted imaging (T2WI) (**Figures 2C, D**). On T2-weighted fat-suppressed imaging, the lesion showed marked signal attenuation, supporting the presence of intralesional fat. In-phase and opposed-phase imaging further confirmed intralesional lipid components (**Figures 2E, F**). Diffusion-weighted imaging (DWI) showed no obvious diffusion restriction, and the corresponding apparent diffusion coefficient (ADC) map showed no signal reduction (**Figures 2G, H**).

Contrast-enhanced imaging: On contrast-enhanced MRI, the lesion showed mild reticular enhancement (**Figures 2I, J**). The mass was closely related to the celiac trunk, superior mesenteric artery, and left renal vein. Based on these findings, a fat-containing tumor was initially suspected, and atypical lipomatous tumor/well-differentiated liposarcoma (ALT/WDL) remained an important differential diagnosis.

After multidisciplinary team discussion, surgical resection of the retroperitoneal tumor was performed. Intraoperatively, the mass was located between the celiac trunk and the root of the superior mesenteric artery, on the left side of the abdominal aorta. The tumor was firm in consistency and showed mild adhesion to the surrounding tissues. Complete excision was successfully achieved. The patient recovered uneventfully after surgery. At the most recent clinical follow-up, the patient was in good general condition, with no definite clinical evidence of recurrence. Given the uncertain long-term behavior of retroperitoneal ASCLT, periodic abdominal CT or MRI surveillance was recommended.

Gross pathological examination revealed a relatively well-circumscribed mass (**Figures 2K, L**). Microscopically, the tumor was composed of mature adipose tissue admixed with spindle cells and collagen bundles in a fibrous to fibromyxoid stroma (**Figure 2M**). The spindle cells showed mild nuclear atypia without marked pleomorphism, tumor necrosis, or brisk mitotic activity. No definite lipoblasts were identified in the examined sections. Immunohistochemical analysis demonstrated positivity for cyclin-dependent kinase 4 (CDK4), partial positivity for murine double minute 2 (MDM2), negativity for P16, and a low Ki-67 proliferation index of approximately 2%. To achieve a definitive diagnosis and exclude atypical lipomatous tumor/well-differentiated liposarcoma (ALT/WDL), fluorescence *in situ* hybridization (FISH) analysis was performed and showed no amplification of the MDM2 gene. Taken together, the histopathological, immunohistochemical, and molecular findings supported the diagnosis of ASCLT.

## 3 Discussion

ASCLT is a rare mesenchymal adipocytic neoplasm. Although it contains atypical spindle cells and may contain pleomorphic adipocytic elements, its biological behavior is generally benign, and the prognosis after local excision is favorable. ASCLT typically occurs in the superficial soft tissues of the extremities and trunk, whereas primary retroperitoneal involvement is exceedingly rare. Reported retroperitoneal cases are limited and heterogeneous: Kirisawa et al. described a 34-mm retroperitoneal tumor in a 74-year-old man that was radiographically suspected to be liposarcoma and showed no recurrence or metastasis one year after resection (4), Bae et al. reported a giant retroperitoneal tumor in an 18-year-old woman, approximately 30 cm on CT and 38 cm grossly, requiring technically demanding complete excision with no recurrence at 12 months (5), and Krupinska et al. reported a retroperitoneal ASCLT in a 62-year-old man and emphasized the importance of immunohistochemical and molecular distinction from other adipocytic neoplasms (3). Compared with these reports, the present case adds detailed CT and multiparametric MRI findings for a small, incidentally detected retroperitoneal lesion adjacent to major abdominal vessels.

On CT, ASCLT may appear as a fat-containing mass with interspersed strands or nodules of soft-tissue attenuation, which may enhance after contrast administration. MRI provides superior soft-tissue contrast resolution and is valuable for diagnosis and differential diagnosis of ASCLT (6, 7). Recent imaging reviews have shown that ASCPLT may present with variable proportions of fatty and non-fatty components and can mimic ALT/WDL, myxoid liposarcoma, dedifferentiated liposarcoma, spindle cell lipoma, or even undifferentiated pleomorphic sarcoma (7). Therefore, imaging findings should be interpreted as suggestive rather than definitive. The adipocytic component typically shows signal intensity similar to subcutaneous fat on both T1WI and T2WI and suppresses on fat-suppressed sequences. By contrast, the spindle cell, fibrous, or myxoid components may show low signal intensity on T1WI, iso- to slightly hyperintense signal intensity on T2WI, and mild reticular or striated enhancement after contrast administration. In the present case, the small lesion size, well-defined margin, focal fat component, absence of diffusion restriction, and mild reticular enhancement favored a low-grade adipocytic neoplasm and were compatible with ASCLT, although pathological and molecular confirmation remained necessary.

A focused comparison with previously reported retroperitoneal ASCLT cases is summarized in **Table 1**. The available cases indicate that retroperitoneal ASCLT may range from a small lesion to a massive tumor occupying the abdominal cavity. Most cases were managed by complete surgical excision because preoperative imaging could not reliably exclude liposarcoma. These observations support multidisciplinary assessment, careful vessel mapping, and postoperative surveillance even when imaging suggests a low-grade lesion.

**Table 1:** Reported retroperitoneal atypical spindle cell lipomatous tumor cases and comparison with the present case.

Reference	Size	Location	Imaging / clinical note	Outcome
Krupinska et al. (3)	About 52 x 17 cm	Large retroperitoneal mass extending into the pelvis	Preoperatively suspected to be liposarcoma	Complete excision; follow-up details discussed in the literature review
Kirisawa et al. (4)	34 mm	Retroperitoneum	Retroperitoneal ASCLT, initially considered a lipomatous tumor	No recurrence or metastasis reported
Bae et al. (5)	About 30 cm on CT; 38 x 24 x 11.5 cm grossly	Giant retroperitoneal mass filling the abdominal cavity and extending into the pelvic cavity	Large fatty mass without adjacent organ invasion	Complete excision; no recurrence or metastasis at 12-month follow-up
Present case	2.8 x 2.0 cm on CT; 2.2 x 2.7 cm on MRI	Between the celiac trunk and the superior mesenteric artery	Small, well-defined fat-containing lesion with mild reticular enhancement and no obvious diffusion restriction	Complete excision; clinically well at the most recent follow-up, with no definite clinical evidence of recurrence; periodic CT or MRI surveillance recommended

The differential diagnosis of ASCLT mainly includes several fat-containing soft tissue tumors, including well-differentiated liposarcoma (WDLPS/WDL), spindle cell lipoma (SCL), atypical lipomatous tumor (ALT), and lipomatous solitary fibrous tumor. These entities differ in biological behavior and management, and careful imaging, pathological, and molecular analysis can help narrow the diagnostic spectrum (**Table 2**).

**Table 2:** Key imaging, pathological, and molecular features for differentiating ASCLT from major fat-containing tumors.

Entity	CT / MRI clues	Pathological clues	Molecular / IHC clues	Clinical implication
ASCLT	Variable fat and non-fatty components; often well-defined; fat suppression confirms lipid; mild reticular or striated enhancement may be seen; diffusion restriction is usually absent or limited	Mature adipocytes admixed with atypical spindle cells in fibrous, collagenous, or fibromyxoid stroma; lipoblasts may be absent or focal; mild to moderate atypia	MDM2 amplification absent; CD34 often positive; RB1 loss may occur; MDM2/CDK4 usually negative or focal	Benign but locally recurrent potential; complete excision and follow-up are appropriate
WDLPS / ALT	Deep or retroperitoneal fat-dominant mass, often large; thick septa and nodular non-fat components; enhancing septa or nodules	Mature adipocytic tumor with atypical hyperchromatic stromal cells in fibrous septa	MDM2 amplification is the diagnostic cornerstone; CDK4 amplification / overexpression is common	Locally aggressive; surgical planning should consider margins and adjacent organs / vessels
SCL	Usually superficial posterior neck, shoulder, or back; variable fat and collagen; generally less aggressive appearance	Bland spindle cells, mature adipocytes, ropey collagen, and mast cells; no significant atypia	CD34 positive; RB1 loss may be present; MDM2 / CDK4 amplification absent	Benign; retroperitoneal location is unusual
Lipomatous SFT	Fat-containing mass with prominent vascularity; heterogeneous signal; moderate to marked enhancement; necrosis or cystic change may occur	Patternless spindle cell tumor with branching vessels and mature adipocytes	STAT6 nuclear positivity and NAB2-STAT6 fusion; CD34 often positive	Usually benign or borderline; vascularity affects surgical planning

ALT, atypical lipomatous tumor; ASCLT, atypical spindle cell lipomatous tumor; CDK4, cyclin-dependent kinase 4; CT, computed tomography; FISH, fluorescence in situ hybridization; IHC, immunohistochemistry; MRI, magnetic resonance imaging; RB1, retinoblastoma 1; SCL, spindle cell lipoma; SFT, solitary fibrous tumor; WDLPS, well-differentiated liposarcoma.

The key CT, MRI, and enhancement differences among these entities are summarized in **Table 3**. ALT/WDLPS is the most important differential diagnosis, particularly in the retroperitoneum. ALT/WDL closely resembles mature adipose tissue but contains fibrous septa with atypical hyperchromatic stromal cells. Imaging features of ALT/WDL usually include a fat-dominant mass with more than 75% adipose content, large lesion size often exceeding 10 cm, thick or nodular septa, and non-adipose nodules (8). Both CT and MRI can demonstrate intralesional fat, whereas MRI is more effective in characterizing septa and non-adipose components. Fibrous septa typically appear hypointense on T1WI and T2WI and may show delayed enhancement. Definitive differentiation relies on pathological and molecular evaluation. MDM2 amplification remains the cornerstone for distinguishing ALT/WDL from ASCLT (9, 10). CDK4 is an important complementary biomarker in liposarcoma biology: CDK4 amplification/expression is common in ALT/WDL and dedifferentiated liposarcoma, has been investigated as a prognostic biomarker, and provides a rationale for CDK4/6 inhibitor strategies in advanced liposarcoma (11, 12). In contrast, ASCLT lacks MDM2 amplification and is typically negative or only focally positive for MDM2/CDK4, as observed in the present case.

**Table 3:** The radiological feature differentiation between well-differentiated liposarcoma, spindle cell lipoma, atypical lipomatous tumor and lipomatous solitary fibrous tumor.

Differential characteristics	CT features	MRI features	Enhancement pattern
Well-differentiated liposarcoma (WDLPS)	Predominantly fat-containing mass with thickened septa and nodular non-adipose components	Fat shows high signal on T1-weighted images; non-fatty components are iso- to hypointense on T1-weighted images and variably hyperintense on T2-weighted images	Mild to moderate enhancement of septa and non-adipose components
Spindle cell lipoma	Well-circumscribed lesion composed almost entirely of fat, without thickened septa or nodular soft-tissue components	Homogeneous high signal on T1-weighted images corresponding to macroscopic fat	Homogeneous high signal on T1-weighted images corresponding to macroscopic fat
Atypical lipomatous tumour (ALT)	Predominantly fatty mass with thickened septa and focal non-adipose components	Similar to WDLPS, with hyperintense fat on T1-weighted images and hyperintense non-fatty areas on T2-weighted images	Mild to moderate, often heterogeneous enhancement of non-adipose components
Fat-forming solitary fibrous tumour	Predominantly soft-tissue mass with variable and usually limited intralesional fat	Fatty areas are hyperintense on T1-weighted images; fibrous components show variable, often relatively low T2 signal	Heterogeneous enhancement of the non-fatty fibrous components

Spindle cell lipoma (SCL) shares morphological features with ASCLT, making the two entities difficult to distinguish on imaging alone. Tumor location and clinical context are helpful. SCL almost exclusively arises in subcutaneous tissues, particularly in the posterior neck and shoulder regions (13), and rarely occurs in deep locations such as the retroperitoneum. Collagen bundles within SCL may show lower signal intensity on T2WI, and enhancement is generally milder than that observed in ASCLT.

Atypical lipomatous tumor (ALT) and WDL represent the same entity at different anatomic sites and should be considered when a fat-containing lesion shows thick septa, non-adipose nodules or masses, infiltrative growth, absence of a capsule, or irregular margins on CT and MRI (14). In deep sites such as the retroperitoneum, the term WDL is generally preferred because complete resection is more difficult and recurrence risk is clinically important.

Lipomatous solitary fibrous tumor is composed of mature adipocytes admixed with bland spindle cells and originates from CD34-positive dendritic interstitial cells. It is considered a benign or borderline tumor and may show fibroblastic, myofibroblastic, or pericytic differentiation, resulting in heterogeneous density or signal intensity. Because of its prominent vasculature and collagen content, myxoid degeneration, necrosis, and cystic change may occur (15–18). Most tumors show moderate to marked arterial-phase enhancement, often with variable central necrosis or cystic areas.

Recognition of these imaging features may influence preoperative management. When a retroperitoneal fat-containing lesion is small, well-defined, shows limited non-adipose nodularity, lacks diffusion restriction, and demonstrates only mild reticular enhancement, ASCLT should be included in the differential diagnosis rather than assuming ALT/WDL. This may prompt multidisciplinary review, consideration of image-guided biopsy or molecular testing when feasible, precise evaluation of the relationship to the celiac trunk, superior mesenteric artery, renal vein, and adjacent organs, and organ-sparing surgical planning. Nevertheless, because imaging overlap with ALT/WDL remains substantial, complete excision and histopathological-molecular confirmation are still required.

## 4 Conclusion

Primary retroperitoneal ASCLT is extremely rare and can mimic ALT/WDL on preoperative imaging. In a retroperitoneal fat-containing mass, small size, well-defined margins, limited non-adipose nodularity, absent diffusion restriction, and mild reticular enhancement may suggest ASCLT and support careful multidisciplinary, organ-sparing surgical planning. Final diagnosis requires histopathological correlation and molecular exclusion of ALT/WDL, particularly by demonstrating the absence of MDM2 amplification, with postoperative surveillance because long-term behavior in this location remains insufficiently defined.

## Data Availability

The original contributions presented in the study are included in the article/supplementary material. Further inquiries can be directed to the corresponding author.
